# Psychological distress in postpartum: influence of late preterm delivery

**DOI:** 10.1186/1824-7288-40-S2-A6

**Published:** 2014-10-09

**Authors:** Zanardo Vincenzo, Francesca Volpe, Rita Maione, Arturo Giustardi, Gianluca Straface

**Affiliations:** 1 Policlinico Abano Terme, Piazza Colombo 1, 35031 Abano Terme, Italy

## Background

Psychological distress in women during the postpartum period has been for a while an issue of great concern. There is substantial evidence that maternal psychological distress after pregnancy is associated with an adverse cognitive and behavioural consequence in the offspring [1]. There are also severe implications during the perinatal period for the mother’s long term mental health [2,3], her partner’s mental health [4,5], and for the parental relationship [6]. The growing trend in late preterm deliveries suggests research on postpartum psychological distress risk in this group of vulnerable women.

## Materials and methods

This prospective case control study was performed with the approval of the ethics committee, in accordance with the Declaration of Helsinki. Women who gave birth from 34/0 to 36/6 weeks and the next women who gave birth from 37^g^ to 40/6 weeks able to give informed consent were eligible.Three days after childbirth, mothers of late preterm infants (n=42) and the next mother of at term infant, matched for parity and delivery route (n=42) completed medical history that covered key demographic and social information and the following questionnaires: State-Trait Anxiety Inventory questionnaire (STAI-Y) [7], Edinburgh Postnatal Depression Scale (EPDS) [8], and Psychological Stress Measure (PSM [9]).

## Results

Findings show that mothers of late preterm infants, presenting with comparable key demographic and social antenatal risk factors, have more stress, anxiety, and depression than mothers of at term infants (State Anxiety-state 42.6±5.3 vs 49.5±9, p<0.0002; Anxiety-trait 39±6.1 vs 45.8±10.1, p<0.02; EPDS 6.3±3.9 vs 9.5±4.5, p<0.008; PSM 38.9±4.5 vs 46±5.9, p<0,001). In addition, Anxiety-state levels were associated with longer time to stay in hospital (days 6.1±1.8 vs 4.7±1.2: p< 0.01).

## Conclusions

These data indicate that late preterm delivered mothers are at increased psychological risk in a critical phase for establish a correct mother infant relationship. This can happen in two ways: first of all perhaps, by averting preterms’ delivery and secondly by working through the distress. Moreover, what should be consider of great importance during the postpartum period is the presence of an entourage that can help relieve the mother from psychological distress and to support her and her child in case of acute symptoms [10]. Taking into account all these consideration it would be great to be able to arrange a psychological treatment for these mothers n terms of their immediate and future well-being. and must therefore be targeted for intervention.
